# Nursing Students’ Perceptions of the Ease of Use and Usefulness of Immersive Virtual Reality Simulation: A Mixed-Methods Study

**DOI:** 10.1177/23779608251356599

**Published:** 2025-07-02

**Authors:** Hege Vistven Stenseth, Marit Hegg Reime, Hilde Sundfær, Camilla Olaussen

**Affiliations:** 1 155319 Department for Postgraduate Studies, Lovisenberg Diaconal University College, Oslo, Norway; 2 Department of Health and Caring Sciences, 1657 Western Norway University of Applied Sciences, Bergen, Norway; 3 Advisor & Head of Department of Educational Support, 155319 Lovisenberg Diaconal University College, Oslo, Norway; 4 155319 Department for Undergraduate Studies, Lovisenberg Diaconal University College, Oslo, Norway

**Keywords:** immersive virtual reality, simulation, nursing education, assessment skills, ease of use, usefulness, mixed methods

## Abstract

**Introduction:**

Nursing education must equip students with skills for systematic patient assessment, using approaches such as the Airway, Breathing, Circulation, Disability, Exposure (ABCDE) assessment and the National Early Warning Score 2 (NEWS2) for early detection of patient deterioration. Immersive virtual reality (IVR) simulations offer a valuable tool for enhancing competency in these two methods.

**Objective:**

This study explored nursing students’ experiences with and perception of the ease of use and usefulness of IVR simulations to develop knowledge and skills of the ABCDE assessment and NEWS2 score and to inform future instructional and pedagogical design.

**Methods:**

A concurrent mixed-methods design was employed. The participants were Bachelor of Nursing students engaged in an IVR simulation focused on developing on ABCDE and NEWS2 Qualitative data were obtained through three focus group interviews involving 18 participants. Quantitative data (*N* = 107) were collected via a postintervention survey. Data analyses were conducted concurrently, and juxtaposed to identify commonalities and discrepancies, with particular attention given to complementary findings.

**Results:**

Four themes were generated through the qualitative analysis: *Navigating Virtual Reality; Bridging Theory and Practice; Empowering Autonomy; and Authenticity and Ambiguity.* Survey results for ease of use indicated high score for ease of use, with average score 4.38 out of 5 (*SD* 0.32). The average usefulness score was 4.40 (*SD* 0.67), implying the IVR simulation as beneficial for enhancing competencies on ABCDE assessment and NEWS2 scoring skills.

**Conclusion:**

Nursing students generally perceived the IVR technology and software as user-friendly and effective in enhancing their knowledge and skills through repetitive practice with a variety of virtual patients. Customizing IVR simulations to align with students’ competency levels is recommended. Future research should investigate the impact of IVR on learners with differing learning styles, experiences, and levels of technological proficiency, and compare its effectiveness to traditional simulations for skill acquisition.

## Introduction/Background

Educational institutions are obligated to prepare students for their future responsibility to provide safe healthcare of outstanding quality (RETHOS, 2019, 2020). Providing safe care and having the ability to identify and respond to patients at risk of clinical deterioration are key competencies that nursing students must cultivate (Oldland et al., 2020; Tirkkonen et al., 2013), and proper training is required in procedures of systematic clinical observation and assessment (McCuistion et al., 2021). Two internationally recognized approaches to systematic clinical observation and assessment are the ABCDE approach (Airway, Breathing, Circulation, Disability, Exposure) and the National Early Warning Score 2 (NEWS2) scoring system (Evans et al., 2021; Mok et al., 2015; Smith & Bowden, 2017). The ABCDE approach systematically and sequentially assesses a patients’ vital systems in a prioritized manner (Soltan & Kim, 2016). The NEWS2 scoring system assesses the degree of severity of illness and risk of deterioration by scoring a patients’ vital signs in a validated scoring system (Welch et al., 2022). The score is based on measurements of respiratory rate, oxygen saturation, body temperature, systolic blood pressure, pulse rate, and the patients’ level of consciousness. The NEWS2 score must be interpreted alongside clinical observations and data gathered through the ABCDE assessment.

One approach to developing these skills is by implementing simulation-based learning (SBL) with immersive virtual reality (IVR). IVR describes a digitally created environment that immersively surrounds users and enhances their sense of presence (SoP) (Bailenson et al., 2008). SBL with IVR simulation offers unique learning opportunities that are not accessible in traditional simulation and skills training (Bauce et al., 2023). By providing access to virtually simulated patients, IVR may enhance nursing students’ opportunities to practice skills (Liu et al., 2023). Gaining insight into how nursing students engage with and benefit from IVR shapes curriculum design and informs evidence-based educational practices and policies that foster competent healthcare professionals.

## Review of Literature

Nurses require robust skills in observation, assessment, interpretation, and analysis to effectively identify and manage deteriorating patients (Mok et al., 2015; Muralitharan et al., 2021). Systematic approaches such as ABCDE assessment and scoring systems such as NEWS2 help nurses detect and respond to changes in patient conditions (Carr et al., 2021; Smith et al., 2013). ABCDE assessment provides a systematic approach to identify change in a patient's condition and to prioritize interventions (Smith & Bowden, 2017). In NEWS2, measurements of vital signs are used to calculate a score that helps assess the patient's clinical risk based on deviations in vital signs (Rigoni et al., 2021). However, nursing students often struggle to gather and assess these data effectively to provide appropriate care (Olaussen et al., 2020; Yilmaz et al., 2015). Tan et al. (2021) identified a need to improve education in physical assessment skills, which aligns with the findings of Schoeber et al. (2022), who report variations in knowledge and skills related to the ABCDE approach among graduated nurses and suggest tailored interventions. According to experiential learning theory (ELT), students learn from experience in a learning process related to the nature of the experience (Kolb, 2014), making active learning with student involvement crucial in nursing education (Kolb & Kolb, 2005; Kolb, 2014). Simulating real-life procedures with virtual patients supports experiential learning through interaction in a virtual world (VW) (Jenson & Forsyth, 2012).

IVR creates a three-dimensional environment that varies in immersion, interactivity, and realism (Chen et al., 2020). Using headsets and hand controllers, students interact with objects with visual and sensory inputs that allow nursing students to experience realistic clinical scenarios (Bauce et al., 2023; Choi et al., 2022) with high SoP in virtual clinical environments (Bradley et al., 2024; Servotte et al., 2020). The level of immersion depends on factors such as graphic quality, perceived realism, interface design, and interaction (Hudson et al., 2019; Servotte et al., 2020). High levels of interactivity in IVR typically enhance SoP, increasing student engagement (Bailenson et al., 2008; Cieslowski et al., 2023; Cypress & Caboral-Stevens, 2022). IVR offers a potential solution to a key challenge in nursing education: the need for direct patient interaction (Liu et al., 2023). Clinical placements may provide limited hands-on practice due to uncertain patient availability and safety concerns.

Challenges with traditional SBL may include high anxiety levels among staff and students, the need for skilled facilitators, and the cost of human patient simulators (HPSs) (Ismail et al., 2024). IVR mitigates these challenges by enabling realistic, interactive learning in a safe and controlled environment with the possibility for individual, self-directed learning (Bodur et al., 2024; Liu et al., 2023). A study by Bodur et al. (2024) found that nursing students had positive attitudes toward IVR simulations and technology, and IVR supported the experience of self-directed learning skills. Bradley et al. (2024) also report a positive perception of IVR for learning. However, they also highlight that IVR imposes a high cognitive load and presents usability challenges. According to Liu et al. (2023), IVR simulations augment the didactic process by enhancing students’ understanding of theoretical concepts. Choi et al. (2022) reviewed experimental studies on SBL with IVR, finding improved outcomes of cognitive and psychomotor skills. In two randomized controlled trials, IVR simulation with nursing students learning the ABCDE approach was not inferior to individual self-practice with physical equipment (Berg & Steinsbekk, 2020, 2021). Qualitative studies of the user experience in nursing education indicate support for using IVR simulations for active learning, practicing and maintaining skills, and enhancing learner motivation (Andreasen et al., 2022; Mäkinen et al., 2023).

Although IVR has demonstrated significant potential in enhancing nursing education by facilitating experiential learning and improving cognitive and psychomotor skills, its integration into curricula remains complex and context dependent. Prior studies have yielded predominantly positive outcomes. However, there remains a limited understanding of how nursing students experience IVR, specifically in the context of learning structured systematic clinical observation and assessment, such as the ABCDE approach and NEWS2 scoring. Furthermore, despite the growing body of research on IVR, variability in student engagement, cognitive load, and usability challenges suggests that its efficacy may not be consistent across learning contexts. To inform future implementation of IVR in nursing education, it is essential to investigate students’ perceptions regarding the usefulness and ease of use of IVR technology and associated software in cultivating specific clinical competencies. Consequently, this mixed-methods study investigates nursing students’ experiences with IVR simulations designed to enhance observational and assessment skills, with a focus on their perceptions of ease of use and usefulness in learning the ABCDE assessment and NEWS2 scoring frameworks.

## Methods

### Design

This study was conducted according to the Mixed-Methods Reporting in Rehabilitation and Health Sciences (MMR-RHS) checklist and standard for mixed-methods reporting (Tovin & Wormley, 2023) and the COonsolidated criteria for REporting Qualitative research: COREQ checklist (Tong et al., 2007). The qualitative data are reported in coherence with the Reflexive Thematic Analysis Reporting Guidelines (RTARG) (Braun & Clarke, 2024). The study employed a concurrent mixed-methods design with parallel qualitative and quantitative data collection and analyses using a side-by-side approach (Creswell, 2022; Creswell & Clark, 2017). This design enables the identification of convergent and complementary findings across the two datasets, providing a comprehensive understanding of students’ experiences and perceptions. The rationale is that the strengths of each method compensate for the limitations of the other (Creswell & Clark, 2017). The aim of the study required prioritizing and weighting the qualitative data to elicit comprehensive descriptions and obtain nuanced insights into the students’ perceptions and experiences (Bradshaw et al., 2017). Qualitative data were generated from focus group interviews, and quantitative data were collected from a descriptive survey. The data were analyzed separately in parallel sequences, after which the two datasets were juxtaposed to identify commonalities or discrepancies, with particular attention given to complementary findings (Creswell & Clark, 2017; Östlund et al., 2011). The analysis and results are presented separately in the Result section along with an overview of complementary data. The Discussion section presents the interpretation and comparison of the findings across the two datasets (Creswell & Creswell, 2023).

### Research Questions

How do nursing students perceive the ease of use and usefulness of IVR simulations in enhancing their knowledge and skills in performing the ABCDE assessment and applying the NEWS2 scoring system?

### The IVR Simulation Intervention

The research was conducted at a university college in Norway. The IVR simulation was integrated into a second-year theoretical course for Bachelor of Nursing students focused on acute care. Participants see the patients from the perspective of their own eyes in the VW. Students participated in groups of 8 to 20, with each group having 90 min to complete the IVR simulations. For the intervention, instructional designers set up the IVR software on 20 PICO headsets with handheld controllers. The software was developed by a Norwegian company specializing in IVR simulations for healthcare; thus, the IVR simulation was presented in the participants’ native language. Participants engaged with IVR technology by donning VR headsets, immersing themselves in a simulated clinical environment designed to replicate the real world. Within this virtual space, they observe and assess virtual patients using hand controllers, viewed as their own hands, to perform physical examinations. The session began with a briefing on learning objectives, session content, and instructions for using the equipment. Students then entered the IVR environment. First, they received audible and visual virtual lectures followed by a multiple-choice test, then entered a virtual hospital room and were instructed to assess four patients. Students then individually randomly selected their patients from a list of names, genders, and ages of 15 available patients. The students continued to perform ABCDE assessments by evaluating airway, oxygen saturation, respiration rate, blood pressure, pulse, level of consciousness, and skin condition following the standardized ABCDE approach. In the NEWS2 program, they calculated NEWS2 scores using provided patient data. Each student received individual textual feedback on the accuracy of their assessments, vital sign measurements, and interpretation of patient data immediately after performance within the IVR technology.

### Sample

This study utilized convenience sampling, with attendance at the intervention serving as the sole inclusion criterion for student participation. All second-year students enrolled in the Bachelor of Nursing program who participated in the IVR simulation (*N* = 107) were eligible to take part in the study. The students received both written and verbal information regarding the study. They were informed that participation could involve completing a survey, attending a focus group interview, or engaging in both activities. All 107 students responded to the survey, and 18 participated in the focus group interviews.

### Data Collection

#### Focus Groups

Three focus group interviews were conducted within 1 week following the IVR simulations, spanning September to November 2022. The first author (MNSc) moderated, with the third (BA) and fourth (PhD) authors acting as assistant moderators. All researchers involved in the study were female and had significant experience in conducting focus group interviews. Each session, lasting 60 min, was held on campus. The number of participants in each interview was, respectively, 8, 6, and 4 students. The participants were encouraged to share their experiences and perceptions without concern for correctness. The researchers remained in a supportive, peripheral role (Krueger & Casey, 2015). All interviews followed the same semistructured interview guide focused on the main categories of interest and in accordance with the TAM model: the perceived ease of use and usefulness of the IVR simulations. The interview guide was developed by the first and fourth authors and discussed with the research team to ensure alignment of the conversations with the aim of the study. Discussions led to only minor semantic alterations (see Interview Guide in the Supplemental material). Open-ended questions were used to elicit diverse perspectives, supplemented by follow-up inquiries to validate the participants’ assertions. Member reflection was practiced during the interviews to gain further insights and provide opportunities for participants to elaborate meanings and topics (Braun & Clarke, 2024). The interviews were audio-recorded for accuracy, and recordings were transcribed verbatim by the first author to ensure reliability.

#### Survey

The survey utilized was an adapted version of the Pediatric Clinical Simulation Survey, originally developed by Verkuyl et al. (2016). It is based on TAM (Davis, 1989, 1993), a broadly used research framework for exploring the ease of use and usability of technology (Atack et al., 2010; Verkuyl et al., 2016). The survey is divided into two sections, corresponding to the two main categories in TAM: perceived ease of use (10 items) and perceived usefulness (6 items) (Verkuyl et al., 2016). Respondents rate the extent to which they perceive ease of use and usefulness using a Likert scale with five response options: (1) strongly disagree, (2) disagree, (3) neutral, (4) agree, and (5) strongly agree. Evidence for the reliability of survey items has been reported in past studies, with Cronbach's alpha results consistently higher than 0.70 (Albu et al., 2015; Verkuyl et al., 2016; Verkuyl et al., 2019). The first and last authors translated the Pediatric Clinical Simulation Survey from English to Norwegian. Certain modifications were made in some of the original survey items to tailor the survey to the IVR simulation. The term *pediatric simulation* was replaced with *IVR simulation*, and *age-appropriate pain management* was replaced with *ABCDE assessment* and *NEWS2 scoring*; also, demographic data were requested. The students completed the survey immediately after the intervention.

### Analysis

In this concurrent mixed-methods study, the analyses of the qualitative and the quantitative data were executed separately in parallel proceedings throughout the analytical phase as outlined by Creswell and Clark (2017). Data generated in the focus groups were analyzed within the methodology of reflexive thematic analysis (RTA) (Braun & Clarke, 2022, 2023, 2024, 2025). The rationale for implementing RTA in this mixed-methods study is that RTA enables a focus on patterns of meaning within an experiential framework to capture and explore subjects’ perceptions and experiences (Braun & Clarke, 2022). This methodology can provide rich, qualitative insights that contextualize and enhance the understanding of quantitative data. The researchers approached the generated data inductively, enabling the capture of participants’ perspectives and understanding within a constructivist theoretical framework (Denicolo et al., 2016). The researchers searched for both semantic and latent meanings throughout the analytic process. The first and last authors performed the analysis through six steps outlined by Braun and Clarke (2022): (1) The transcripts were read and reread repeatedly to acquaint the researchers with the generated data. Both authors took notes on their initial reflections and assumptions and then reflected on and questioned them in joint discussions, striving to acknowledge and reflect on their assumptions before proceeding with the analysis (Braun & Clarke, 2022). (2) The initial coding process involved systematic and comprehensive coding of semantic and latent features of the participants’ statements pertinent to the research objectives. (3) The authors examined the codes for patterns and meanings. Both authors generated potential themes by grouping clusters of codes that shared similar meanings or concepts. (4) The initial themes were reviewed by examining both the coded extracts and the entire dataset to ensure that themes accurately captured the main patterns present throughout the dataset. (5) Themes were defined through an iterative process of discussion and reflection among the authors, with the second author also participating. Subsequently, four themes were generated and named. (6) Ultimately, the first author produced an analytic text on the thematic content to address the aim of the study. All authors engaged in reflexive discussion in finalizing the analytic text. Table 1 provides two examples of the analytic process from codes to themes.

**Table 1.** Example of the Analytic Process of Reflexive Thematic Analysis.

Theme	Subtheme	Codes
Navigating Virtual Reality	Physical and psychological impacts of being in the virtual world Support in the process of learning with new technology	Physical symptoms Being in a different reality Another world Unusual experience Adopting new technology Support and help
Bridging Theory and Practice	Developing knowledge and skills Need for advanced interaction and increased complexity	Repetitive training Available learning situations Access to training Need for challenge Wish for more complexity

Data from the survey were analyzed using IBM SPSS Statistics v. 28.0 (IBM Corp., Armonk, NY). Descriptive statistics were used to calculate item means (on a scale from 1 to 5) and standard deviations (*SD*s). The respondents’ overall mean scores within the sections “Ease of use” and “Usefulness” were calculated by averaging the score of all items within each of the two sections. Higher scores indicate greater levels of agreement, while lower scores indicate levels of disagreement. The internal consistency of the overall survey and its two sections, ease of use and usefulness, was described by Cronbach's alpha values.

## Research Ethics

The university college and the Norwegian Agency for Shared Services in Education and Research (SIKT) approved the study (ref. number: 215937). The study adheres to the principles of the Declaration of Helsinki (World Medical Association, 2013). Participation was voluntary, requiring written informed consent for both the focus group interviews and the survey. The consent forms clearly outlined the participants’ rights, and information was reiterated verbally before participation, including the option to withdraw at any time. All data were deidentified and anonymized to ensure confidentiality. The researchers had no supervisory or assessment roles vis-à-vis the participating students, ensuring impartiality.

## Results

The survey was completed by 107 students, of whom 18 also participated in focus group interviews. Table 2 presents the participants’ demographic variables.

**Table 2.** Demographic Variables of the Participants.

Focus groups	Group 1	Group 2	Group 3	Total
Participants (*n*)	8	6	4	18
Female (*n*)	6	6	4	16
Male (*n*)	2	0	0	2
Age: mean (range)	23.4 (20–27)	22.5 (20–33)	21.3 (21–22)	22.6 (20–33)
**Survey**				
Participants (*N*)				107
Female (*n*)				96
Male (*n*)				11
Age: mean (range)				23.64 (19–50)
Prior experience with immersive virtual reality technology (*n*)				29

## Qualitative Data

The qualitative data analysis generated four themes: Navigating Virtual Reality; Bridging Theory and Practice; Empowering Autonomy; and Authenticity and Ambiguity. In the quotations below, each respondent (R) and focus group (FG) is identified by number.

### Navigating Virtual Reality: Interaction With Technology and Software in IVR Simulations

Most students found the technical equipment intuitive and easy to use. The graphics and interfaces in the ABCDE assessment and NEWS2 scoring programs were considered superior in quality to other IVR programs they had encountered. The students perceived the staff's presence and the comprehensive instructions as reassuring and providing a sense of security, enabling them to fully engage in the IVR simulation. One student remarked, “Using the technology went quite smoothly. It was, however, good to have assistance, because it can be scary to use new equipment in case you suddenly break something” (R4, FG2). Another commented, “I found it very easy to navigate, as I always knew what I was supposed to do … and it was easy to use the controls, and they vibrated nicely when I got close to something” (R8, FG3). Several students encountered problems with incorrect game boundaries, which impaired their ability to assess the patient or access virtual equipment, potentially stalling the learning process without assistance. One said, “I didn’t quite know what to do to solve [the boundary problem]. So, having people who could help me when I didn’t understand what to do myself was nice” (R2, FG 2).

The students generally reported adapting quickly to the IVR environment without anticipating difficulties, confusion, or fear within the VW. However, the concept of existing simultaneously in parallel worlds—both the physical world and the VW—evoked apprehension, loss of control, and anxiety in some students. One shared, “I was a little … like … nervous beforehand … because you stand in one room, and then you get pulled into another room. To know that you see that you are in a room … but you are not really there” (R3, FG3). Another student explained, “Since you are not there [in the VW] for real, you cannot control your own body, you cannot cover your own vision” (R5, FG2). A subset of students reported physical discomfort during and after the IVR simulation, with common complaints including headache, dizziness, ocular strain, and fatigue. If not addressed, these discomforts and fears may potentially impact both ease of use and overall usability in educational settings. Despite these challenges, most students completed their assigned tasks, citing the excitement of the experience as a motivating factor. One student remarked, “For me, IVR was very tiring, because I got a lot of pain and felt my eyelids were heavy, as they were being pulled down constantly. The fact that it was exciting made me endure longer” (R1, FG3).

### Bridging Theory and Practice: Enhancing Nursing Competencies Through IVR Simulations

Students with theoretical knowledge of the ABCDE assessment and NEWS2 perceived the IVR simulation with repetitive training through sequential procedural enactment to be highly valuable for translating theoretical knowledge into practical nursing skills. One student explained, “It's good to be able to practice skills [in] ABCDE and NEWS2, because when you only read about it and then must do it with real patients—that is a problem. With the IVR simulation, you avoid being so stressed when you do it in placement settings. You can do it because you have practiced it, even without a real patient” (R6, FG1). Most students agreed that IVR simulations had the potential to prepare them for situations they may encounter in their future work as nurses. As one noted, “We have learned about these procedures as NEWS2, but getting it done was something else. It became more real, even if it was not real. One understands more how to use the procedures. Good for both patients and us” (R1, FG1).

Students with prior experience in ABCDE assessment and NEWS2 scoring observed that the IVR simulation was suited to a lower competency level than their current proficiency, making it more apt for the learning needs of first-year nursing students. One said, “I think it was a bit too easy. In ABCDE, what we did was basic. I feel I would have learned more from it if we had to draw in a bit more … [assessment]. But for the first-year students, it would have been very good” (R3, FG2). Several students advocated for a tiered structure with progressive levels of complexity, opportunities for decision making, and initiation of nursing actions, with feedback provided through a dynamic progression of patient conditions in the IVR simulation programs. One explained, “I think it would have been nice to have different levels of difficulty that you could choose from in the IVR simulation. Choosing the level that I want to start with would have allowed me to climb up levels at my own pace” (R6, FG2). They suggested that this approach would better facilitate enhanced nursing competencies and preparedness for future professional nursing responsibilities.

### Empowering Autonomy: Student Independence and Engagement in IVR Simulations

Several students expressed a sense of autonomy and independence during their learning experience in the IVR simulation, which motivated them. This autonomy allowed them to focus on individualized learning, fostering increased concentration and engagement. As one student explained, “When you put the IVR headset on, you are in control of what you are doing. It is your own world. No one else is paying attention to whether you are doing wrong or right … It was nice to just focus on myself instead of thinking about what others around me can see” (R2, FG3)*.* Students perceived the opportunity to perform individually in IVR simulation as an opportunity to practice future responsibilities as nurses and take charge of patient care without the immediate oversight of clinical supervisors or educators. They appreciated being able to practice ABCDE assessment and NEWS2 scoring autonomously, describing it as a positive, liberating experience. Across the focus groups, the students emphasized the advantage of the accessible, flexible training offered by the IVR simulation, which allowed for independent practice without reliance on peers or instructors. Additionally, some students expressed a desire for increased access to IVR equipment, enabling further self-directed skills practice.

### Authenticity and Ambiguity: Student Perceptions of Realism in the IVR Simulations

Most students found the virtual patients to be realistic, and they naturally assumed the role of a nurse upon entering the virtual patient room, responding to patients as though they were real. One student shared, “My patient coughed, so every time the patient raised his elbow and coughed, I kind of jumped back” (R5, FG2). Another stated, “I measured the pulse of the patient. I made eye contact with the patient, and then I smiled at him. I found it very realistic” (R8, FG1). A significant advantage underscored by some students was the IVR simulation's ability to facilitate detailed observation and assessment of deviations in vital parameters. All the focus groups discussed differences between IVR simulation and alternative learning methods, such as traditional simulation and role-play. The students appreciated that IVR simulation allowed them to encounter a variety of patients with diverse clinical deviations in a single learning activity. One explained, “When we use each other as patients in physical simulation, it becomes more drama, and it is difficult to observe deviations in NEWS2 or ABCDE. Deviations can be observed clearly in IVR simulation, whereas in role-play we must pretend” (R2, FG3).

Some students expressed concerns about the lack of variety and nuance in the virtual patients’ reactions, which could lead to potential confusion or errors in assessment. One student shared, “I found it difficult to assess the patient's skin when performing ABCDE. If it was a bit of an odd color on the skin, I thought it could be a wound. But I was not sure, and it could be wrong” (R3, FG1). Some also noted challenges in performing the hands-on actions of clinical tasks. For instance, students had difficulties in accurately perceiving pulse variations and gaining full access to the patient's body. One student described the challenge of airway assessment: “It was difficult to assess airways … I was a bit short, so I couldn’t see into their mouths … I had to stand on my tiptoes … so it was a bit challenging” (R8, FG2). Many students expressed a desire for more interaction and communication opportunities with virtual patients to enhance the realism of the interaction in the experience. A small number of students hesitated to fully engage with the IVR simulation, citing difficulties related to the perceived lack of authenticity in virtual patients.

### Quantitative Results

All 107 students completed the survey, yielding a 100% response rate. The overall internal consistency of the survey, as measured by Cronbach's alpha, was 0.82. At the section level, both the “Ease of Use” and “Usefulness” subscales demonstrated satisfactory reliability, with Cronbach's alpha values of 0.91 and 0.73, respectively, both exceeding the commonly accepted threshold of 0.70. The survey employed a five-point Likert scale from 1 (strongly disagree) to 5 (strongly agree), with 3 representing a neutral response. The responses were modestly skewed toward the “strongly agree” end of the scale. In the “Ease of Use” section, the overall mean score among students was 4.38 (*SD* = 0.32) out of a maximum of 5, indicating that students generally found the ABCDE and NEWS2 IVR simulation easy to use. This finding suggests minimal issues related to technical functioning, graphical interface, or overall user experience with the technology utilized in the simulation. However, it is noteworthy that, at the item level, the statement “I did not have any technical problems using the IVR simulation” received the lowest mean score in this section (mean < 4), indicating that a subset of students encountered technical difficulties during the simulation (see Table 3).

**Table 3.** Students’ Survey Responses to the ABCDE and NEWS2 IVR Simulations Ease of Use and Usefulness (*N* = 107).

Survey sections, ease of use and usefulness, with corresponding items **(Likert scale** from 1 = strongly disagree to 5 = strongly agree)	Mean (*SD*)	Range
**Ease of use (overall)**	**4.38** (**0.32)**	**2.9**–**5.0**
1. It was easy to use the IVR simulation	4.60 (0.76)	1.0–5.0
2. I wanted to continue working through the IVR simulation	4.29 (1.12)	1.0–5.0
3. The text information on the screen was clear	4.81 (0.50)	2.0–5.0
4. The text information on the screen was easy to read	4.04 (1.12)	1.0–5.0
5. I found it easy to know what to do at each stage of the IVR simulation	4.68 (0.75)	2.0–5.0
6. I did not have any technical problems using the IVR simulation	3.67 (1.55)	1.0–5.0
7. The visual quality of the graphics in the IVR simulation was good	3.93 (1.11)	1.0–5.0
8. The situations presented seemed realistic	4.60 (0.64)	2.0–5.0
9. The pace of the “action” in the IVR simulation was good	4.51 (0.68)	2.0–5.0
10. The audio quality in the IVR simulation was good	4.63 (0.64)	2.0–5.0
**Usefullness (overall)**	**4.40** (**0.67)**	**2.3**–**5.0**
11. I think the IVR simulation will help students to perform better ABCDE assessments and NEWS2 scores of patients	4.62 (0.70)	2.0–5.0
12. I think the IVR simulation helped me develop skills in doing the right assessments of patients	4.44 (0.78)	2.0–5.0
13. The IVR simulation will be a useful addition to clinical experiences for students	4.70 (0.57)	2.0–5.0
14. I think the IVR simulation improved my knowledge regarding ABCDE assessment and NEWS2 scoring	4.32 (0.88)	2.0–5.0
15. I think the IVR simulation helped me develop my skills regarding ABCDE assessment and NEWS2 scoring	4.30 (0.83)	2.0–5.0
16. I think the IVR simulation helped me develop my ability to prioritize care	4.01 (1.00)	1.0–5-0

*Note*. ABCDE= Airway, Breathing, Circulation, Disability, Exposure; IVR= Immersive virtual reality; NEWS2= National Early Warning Score 2; SD = standard deviation.

The overall mean score in the “Usefulness” section of the study was 4.40 (*SD* 0.67), implying that the students regarded IVR simulation as a beneficial method for practicing ABCDE assessment and NEWS2 scoring skills (Table 3). Although still relatively high (mean > 4), the lowest item level score in this section was observed for item 16, “I think the VR simulation helped me develop my ability to prioritize care.” This suggests that students perceived the ABCDE and NEWS2 IVR simulation as somewhat less effective in enhancing their prioritization skills compared to other aspects of the training. Table 3 presents the mean score and range of students’ responses regarding the perceived ease of use and usefulness of the IVR simulation.

### Integration of Qualitative and Quantitative Results

The researchers compared the qualitative data and quantitative results using side-by-side comparison (Creswell & Creswell, 2023). Table 4 provides a visual overview and combined display of the complementary findings from the qualitative and quantitative analyses.

**Table 4.** Coinciding and Complementary Findings of the Qualitative and Quantitative Data.

Qualitative findings	Quantitative findings (N = 107) (Likert scale from 1=strongly disagree to 5=strongly agree)		
Navigating virtual reality: Interaction with technology and software in IVR simulations *	Survey items**	Mean (*SD*)	Range
The technical equipment was intuitive and user friendly, facilitating easy navigation within the virtual environment The graphics and interfaces of the programs in the IVR simulation were generally considered of good quality	1. It was easy to use the IVR simulation 5. I found it easy to know what to do at each stage of the IVR simulation 6. I did not have any technical problems using the IVR simulation 7. The visual quality of the graphics in the IVR simulation was good 9. The pace of the “action” in the IVR simulation was good	4.60 (0.76) 4.68 (0.75) 3.67 (1.55) 3.93 (1.11) 4.51 (0.68)	1.0–5.0 2.0–5.0 1.0–5.0 1.0–5.0 2.0–5.0
Staff assistance promoted security and empowerment for students	6. I did not have any technical problems using the IVR simulation	3.67 (1.55)	1.0–5.0
Despite psychological and physiological challenges, most students were motivated to complete the task	2. I wanted to continue working through the IVR simulation	4.29 (1.12)	1.0–5.0
**Bridging theory and practice: enhancing nursing competencies through IVR simulations**	**Survey items**		
IVR simulations have the potential to prepare students for real-world nursing situations Students valued the opportunity to transfer theoretical knowledge to nursing actions in a safe context	11. I think the IVR simulation will help students to perform better ABCDE assessments and NEWS2 scores of patients 13. The IVR simulation will be a useful addition to clinical experiences for students	4.62 (0.70) 4.70 (0.57)	2.0–5.0 2.0–5.0
Students advocated for tiered complexity levels to accommodate individualized learning experiences	16. I think the IVR simulation helped me develop my ability to prioritize care	4.01 (1.00)	1.0–5.0
**Empowering autonomy: student independence and engagement in IVR simulations**	**Survey items**		
Students explored their nursing responsibilities without being observed or assessed Autonomy and independence in IVR simulation allowed individual learning and a focus on individual learning needs	14. I think the IVR simulation improved my knowledge regarding ABCDE assessment and NEWS2 scoring 15. I think the IVR simulation helped me develop my skills regarding ABCDE assessment and NEWS2 scoring	4.32 (0.88) 4.30 (0.83)	2.0–5.0 2.0 5.0
The perceived excitement of the experience was a motivating factor	2. I wanted to continue working through the IVR simulation	4.29 (1.12)	1.0–5.0
**Authenticity and ambiguity: student perceptions of realism in the IVR simulations**	**Survey items**		
Virtual patients were generally perceived as realistic Some limitations were noted in patient reactions and interaction opportunities	7. The visual quality of the graphics in the IVR simulation was good 8. The situations presented seemed realistic	3.93 (1.11) 4.60 (0.64)	1.0–5.0 2.0–5.0

*Note*. * Generated theme from the qualitative analysis; presented under Qualitative Data heading.

** Survey items: refer to results presented in Table 3.

ABCDE= Airway, Breathing, Circulation, Disability, Exposure; IVR= Immersive virtual reality; NEWS2= National Early Warning Score 2; SD = standard deviation.

## Discussion

This study explored nursing students’ nursing students’ perceptions on the ease of use and usefulness of IVR simulations for enhancing their knowledge and skills in performing the ABCDE assessment and applying the NEWS2 scoring system. The IVR simulation was widely perceived as a positive, valuable addition to other learning activities. Overall, they reported the IVR technology to be intuitive and easy to use, although having staff present for technical support gave an enhanced sense of security. For the usefulness of IVR simulation, students perceived it as a valuable and engaging complement to traditional simulation methods and real-life patient interactions in developing knowledge and skills in ABCDE assessment and NEWS2 scoring. They also pointed at several points for improvement. The following section will compare and discuss the interesting findings from the qualitative data and quantitative results with other relevant published research.

### Technological Proficiency, Engagement, and Impact in IVR Simulations

Technological proficiency is a crucial competency for future nurses, and a lack of it may negatively impact the quality of care provided (Nes et al., 2021). The quantitative findings show high scores for the items on ease of navigation and sequential progress in the IVR simulation as well as for the item “It was easy to use the IVR simulation.” However, the item “I did not have any technical problems using the IVR simulation” presented he lowest mean score, indicating that several students encountered a technical issue during the IVR simulation. This is also validated through a large standard deviation. In accordance with the findings of several other studies (Bodur et al., 2024; Bradley et al., 2024; Mäkinen et al., 2023), the qualitative findings indicate that students found the technology intuitive and easy to use, and although some encountered technical challenges, the overall impression was positive for ease of use. One factor possibly influencing the students’ positive response to the technology's quality and accuracy was their access to technical support. Also, Mäkinen et al. (2023) and Shorey and Ng (2021) found that technical assistance is essential when introducing new learning technologies so as not to impair student learning by a shift in focus from the IVR simulation to problems with the technology. Nursing students must become proficient not only in navigating the VW but also in mastering the necessary technical skills, which is a key consideration in integrating IVR simulation into nursing education. Notably, Kardong-Edgren et al. (2019) found that students who self-identified as gamers adapted more rapidly to the IVR environment and were able to focus on the content of the simulation with greater ease. Experience with technology and technological proficiency may facilitate the use of IVR technology for educational purposes. This is an important point to consider when implementing IVR simulations, and the notion aligns with ELT (Kolb, 2014) and with learning from experience to conceptualize and transfer knowledge and skills to other settings.

The study's qualitative data indicate that most students found the IVR simulation exciting, which prompted its completion despite some experiences of distressing symptoms. The quantitative data complement this finding with a high mean score for item 2: “I wanted to continue working through the IVR simulation.” This suggests that the students were engaged and motivated to complete the learning activity. This aligns with the findings of Shorey and Ng (2021), who report that nursing students are interested in IVR and motivated to learn within the virtual environment, as they perceive IVR to be entertaining and enjoyable. The novelty of new technologies may foster engagement and excitement, which in turn may enhance motivation and enjoyment, potentially facilitating learning (Parong & Mayer, 2018, 2021). This accords with ELT, which contends that students must actively engage to acquire and construct new knowledge (Kolb, 2014). However, the substantial standard deviation and the full range of responses (1–5) indicate considerable variability in participants’ perceptions. While most students rated the experience positively, the observed dispersion suggests that some students may benefit more from alternative teaching strategies or may require additional support to engage effectively with the technological aspects of the learning environment. Addressing this variability is essential to ensure equitable opportunities for all students to optimize their learning outcomes.

Consistent with the findings in this research, several studies report that students may experience cybersickness during and/or after IVR simulations. Cybersickness can manifest a range of physical and emotional symptoms, including headaches, eye strain, fatigue, and dizziness (Lavoie et al., 2021; Servotte et al., 2020). Cybersickness may impair the learning experience and quality of learning by IVR simulation, as reported by a subset of the students in the current study. According to Dubovi (2022), IVR environments are highly stimulating both cognitively and emotionally, but some evidence suggests that high levels of immersion in IVR simulation cause high cognitive load, which may lead to decreased learning (Bradley et al., 2024; Dubovi, 2022; Parong & Mayer, 2021). Although existing research indicates that IVR simulations can enhance student engagement and motivation, factors that may negatively impact learning, such as cybersickness and reduced engagement, warrant further investigation.

### Perceived Usefulness for Nursing Education

As suggested in prior studies, both the qualitative and quantitative findings of the current study support IVR simulation's potential as a pedagogical tool in nursing education for enhanced knowledge and skills in ABCDE assessment and NEWS2 scoring procedures (Berg & Steinsbekk, 2020, 2021; Bodur et al., 2024; Choi et al., 2022; Liu et al., 2023). The qualitative data suggest that students gained knowledge and skills through sequential repetitive training in the IVR programs and the opportunity to apply theoretical knowledge in practical training. In the survey results, students gave high scores on knowledge and skills related to ABCDE assessment and NEWS2 scoring as well as to the transferability of knowledge and skills to clinical situations. The complementary results indicate that the students valued the IVR simulation as a useful addition to clinical experience for nursing students. The qualitative findings also suggest the importance of being able to apply knowledge and practice skills in “real” patient situations in the IVR simulations. According to Kolb (2014), students must be actively involved to obtain knowledge through experience, because theory without experience does not reshape understanding of a subject matter.

An unexpected finding in the study was the absence of student discussions regarding the lack of a hands-on or tactile feel when interacting with virtual patients, despite their nonphysical nature. This contrasts with the study by Bradley et al. (2024), which identified the lack of tactile elements as experienced in real-life simulations as a notable drawback for usability. Nevertheless, our findings align with previous research, indicating that IVR simulations offer realistic, interactive learning experiences and may be regarded as a hands-on approach within the VW (Bodur et al., 2024; Liu et al., 2023). Concurrently, it is important to acknowledge that some students described challenges in perceiving virtual patients as authentic. This may be related to the score on item 7: “The visual quality of the graphics in the IVR simulation was good,” (3.93), with a standard deviation of 1,11, and range from 1 to 5, but may also be related to technical issues. Students’ abilities to create narratives and form mental representations of the virtual environment may influence their perceived SoP, which in turn affects their engagement in the experience. Perceived realism is believed to be related to SoP and can impact learning outcomes (Servotte et al., 2020). While SoP and the engagement of multiple senses and emotions are believed to enhance learning (Bauce et al., 2023), they can also hinder the learning process by imposing a heavy cognitive load (Bradley et al., 2024; Dubovi, 2022).

An overall discovery across the findings was that the qualitative findings provide a more in-depth description of certain shortcomings of the intervention, particularly regarding its ability to stimulate students’ problem-solving skills, as they expressed a desire for more complex cases. Although the quantitative data suggest that most students perceived that they improved their knowledge and developed skills regarding ABCDE assessment and NEWS2 scoring, the qualitative findings elaborate that students held mixed opinions of the IVR simulation's perceived level of difficulty and complexity and the need to be challenged. This aligns with the work of Kolb and Kolb (2005) and Kolb (2014), which emphasizes the importance of integrating pre-existing knowledge with information gained through concrete experiences to create new knowledge and understanding. The current study indicates that balancing the needs of students with diverse levels of prior knowledge and experience will be valuable in meeting learners’ needs by optimizing the level of challenge in IVR simulations for nursing education. Duncan et al. (2012) and Fowler (2015) identify several variables that need to be aligned with students’ competency levels, including the specific student group, educational activities, learning theories, the learning environment, and supporting technologies. This is consistent with the observations of Radianti et al. (2020), who note that evaluations of IVR applications in education have focused primarily on usability rather than on learning outcomes. This highlights the need for pedagogical strategies aimed at optimizing IVR simulation in nursing education. Bauce et al. (2023) further highlight the limited application of theoretical frameworks in guiding the implementation of IVR in nursing education. They underscore the need for a robust theoretical foundation to support pedagogical integration, addressing various learning domains to promote enhanced knowledge and skills. Findings in this study indicate that IVR simulation software in nursing education should be customized to match learners’ competency levels appropriately. Additionally, providing options for learners to select the level of difficulty and complexity according to their individual learning needs is essential.

### Strengths and Limitations

This study utilizes a robust methodology, integrating both quantitative and qualitative data. The agreement observed between both data sets enhances credibility and strengthens validity of the research data (Creswell, 2022; Creswell & Clark, 2017; Hashemi & Babaii, 2013). The design facilitated a comprehensive understanding of the research topic, ensuring rich, diverse data in addition to enabling triangulation of the findings (Östlund et al., 2011). Several measures were implemented to increase trustworthiness and rigor of the qualitative data. The research process was meticulously documented to promote dependability. The coding structure, initial and defined themes, and the analytic text were reviewed iteratively. Reflexivity was maintained through the authors’ continuous questioning and reflecting on codes, themes, and text, while considering their subjectivity, values, and contributions to their interpretations in joint discussions (Braun & Clarke, 2022, 2023). This process strengthens the credibility of the data generated. Detailed information about the intervention, research context, and participants were provided to enhance transferability. The quantitative sample size was sufficient to ensure statistical power.

However, some limitations must be acknowledged. The use of convenience sampling from a single nursing education institution and a single educational intervention may limit transferability. Qualitative data were generated through focus groups which may not have captured all perspectives, and there is a risk that students with more positive attitudes volunteered for the focus groups. Despite this, the complementary alignment between the qualitative and quantitative data suggests minimal influence. There is a risk of confirmation bias in selecting complementary findings from the qualitative and quantitative data, as the methods may have addressed different aspects of the phenomenon (Hussein, 2009). To mitigate this, the authors practiced reflexivity and iterative analysis and used investigator triangulation to strengthen credibility (Krueger & Casey, 2015). Both methodological and analytical triangulation enhanced validity and completeness (Hussein, 2009). Limitations also include potential reactive validity bias due to the novelty effect of IVR technology (Onwuegbuzie & Leech, 2007). The researchers’ multifaceted roles in conducting the intervention, focus group interviews, and survey recruitment may have influenced the data. Additionally, the interview guide was not piloted, and the original survey was translated into Norwegian without back-translation, which may have affected data clarity and consistency.

### Implications for Practice

The study's results underscore key considerations for integrating IVR technology into nursing education. IVR simulations effectively support skill development in ABCDE assessment and NEWS2 scoring, providing students with valuable opportunities for repetitive, realistic practice. For successful implementation, educators should align technology with students’ competency levels and offer sufficient technical support to address challenges. Additionally, insights into student experiences with IVR can inform future software and simulation design enhancements, optimizing user engagement and educational outcomes.

## Conclusions

Findings indicate that students perceived the IVR technology, the ABCDE assessment, and the NEWS2 scoring program as easy to use, featuring intuitive interfaces that may have enhanced learning by enabling them to concentrate on mastering nursing procedures rather than grappling with complicated technology. The technical support provided by the faculty was valued and deemed necessary for success. The IVR technology and programs were perceived as useful for gaining knowledge and skills through sequential, repetitive learning with realistic virtual patients who deviated from normal physical conditions. However, the learning activities must be designed for the proper proficiency level and aligned with learning objectives and learning outcomes. Future research should investigate how IVR simulation affects learners with diverse learning styles, experiences, or technological proficiencies as well as the factors contributing to enhanced learning in IVR simulations. Nursing education would benefit from research on how IVR simulations differ from traditional simulations in terms of skill acquisition. Also, there is a need to explore the motivational aspects of IVR technologies and programs, especially regarding the novelty aspect after IVR technology has transitioned from a novelty to a more familiar learning tool.

## Supplemental Material

sj-docx-1-son-10.1177_23779608251356599 - Supplemental material for Nursing Students’ Perceptions of the Ease of Use and Usefulness of Immersive Virtual Reality Simulation: A Mixed-Methods Study Supplemental material, sj-docx-1-son-10.1177_23779608251356599 for Nursing Students’ Perceptions of the Ease of Use and Usefulness of Immersive Virtual Reality Simulation: A Mixed-Methods Study by Hege Vistven Stenseth, Marit Hegg Reime, Hilde Sundfær and Camilla Olaussen in SAGE Open Nursing
