# Supplementary material for: Nursing Students’ Perceptions of the Ease of Use and Usefulness of Immersive Virtual Reality Simulation: A Mixed-Methods Study
Source: SAGE Open Nurs. 2025 Jul 2;11:23779608251356599. doi: 10.1177/23779608251356599 (PMC12227877; doi:10.1177/23779608251356599)
Supplement: sj-docx-1-son-10.1177_23779608251356599 - Supplemental material for Nursing Students’ Perceptions of the Ease of Use and Usefulness of Immersive Virtual Reality Simulation: A Mixed-Methods Study [file sj-docx-1-son-10.1177_23779608251356599.docx]

| **Ease-of-Use:** | **Follow-up** |
| --- | --- |
| **1. You have diverse experience with IVR technology: What are your perceptions and experiences of using this technology and this particular software for the ADCDE assessment and NEWS2?** | - What were the easy aspects of using the technology?  -What aspects of the software did you find most helpful for understanding ABCDE assessment and NEWS2 scoring?  - What were the challenging aspects of using the technology?  - Were there any features or functionalities you found confusing or difficult to use?  - How did the use of this technology affect your engagement or motivation to learn?  - Would you be able to, and is it likely that you would use the technology on your own without technical assistance if provided the chance? |
| **2. How did you experience being in the virtual world?** | - How did you experience performing observations and assessments in the virtual world?  - How did you experience interacting with the virtual patient?  -Were there any moments when you felt disoriented, distracted, or particularly engaged?  -What are your perceptions of the virtual patient?  -What suggestions would you have for improving the virtual experience? |
| **Usability:** |  |
| **1. How would you evaluate the utility value of carrying out the IVR simulations?** | - Which aspects contributed to learning?  - Which aspects inhibited learning? |
| **2. What are your perceptions of the relevance of the IVR simulations on ABCDE assessment and NEWS2 scoring in relation to your current level of nursing education?** | - In what ways did the simulations feel appropriate or not for your current knowledge and skill level?  - Did the simulations challenge you in a way perceived as useful for learning? |
| **3. How well do you think these simulations align with the content and learning objectives of the courses you are currently attending?** | - Were there any specific course topics or objectives that were directly supported by the simulation experience?  - Did the simulation help you prepare for any assessments, skills labs, or clinical placements? |
| **4. What are your reflections on the possibilities and the limitations of using IVR technology and the current software?** | - How do you think the use of IVR could be better integrated into your nursing curriculum?  - Do you think the simulation helped bridge the gap between theory and practice? Why or why not? |
